# The economic burden of rotavirus infection in South Korea from 2009 to 2012

**DOI:** 10.1371/journal.pone.0194120

**Published:** 2018-03-19

**Authors:** Kyung Suk Lee, Ye-Rin Lee, So-Youn Park, In-Hwan Oh

**Affiliations:** 1 Department of Pediatrics, CHA Bundang Medical Center, CHA University School of Medicine, Seongnam, Korea; 2 Department of Preventive Medicine, Graduate School, Kyung Hee University, Seoul, Korea; 3 Department of Medical Education and Humanities, School of Medicine, Kyung Hee University, Seoul, Korea; 4 Department of Preventive Medicine, School of Medicine, Kyung Hee University, Seoul, Korea; New York City Department of Health and Mental Hygiene, UNITED STATES

## Abstract

Rotavirus is a common cause of diarrhea worldwide, and vaccination prevents rotaviral gastroenteritis. Since the rotavirus vaccine was introduced in Korea in 2007, the prevalence of rotaviral gastroenteritis has decreased. However, little is known on the economic burden of rotavirus infection and its variations in Korea. Here, we estimated the economic costs of rotavirus infection from 2009 to 2012 using nationwide data from the National Health Insurance Service (NHIS) claims. Socioeconomic costs were subdivided into direct and indirect and measured with a prevalence-based approach. Costs were converted from Won to United States dollars (US$). The number of children <5 years old infected with rotavirus decreased from 21,437 to 10,295 during the study period, representing a decrease in prevalence from 947 to 443 per 100,000. The sum of direct and indirect costs also decreased, from $17.3 million to $9.6 million, and the days of admission decreased from 76,000 to 38,000. However, per capita expenditures slightly increased, from $809 to $934. Thus, the economic burden of rotavirus infection decreased after implementation of rotavirus vaccination. Including the vaccine as part of the national essential vaccination program could reduce the prevalence of and economic loss from rotavirus infection in Korea.

## Introduction

Rotavirus (RV) infection is the most frequent cause of diarrhea in young children worldwide [1]. It is estimated to cause around 2 million hospitalizations and 440,000 deaths of children <5 years old annually worldwide [2]. Before the introduction of the RV vaccine in Korea, among children <5 years old, the incidence of RV infection was 56.9 cases per 1,000 children, and hospitalizations due to RV diarrhea were 11.6 cases per 1,000 children from 2002–2004 [3].

Two RV vaccines are currently administered to children globally, RotaTeq ([RV5]–Merck and Company, Whitehouse Station, NJ) and Rotarix ([RV1]–GlaxoSmithKline Biologicals, Rixensart, Belgium) [4–6]. RV5 is a live pentavalent RV vaccine derived from human-bovine strains (G1, G2, G3, G4, and P[8]), and it is administered at 2, 4, and 6 months of age [4–6]. RV1 is a live monovalent RV vaccine derived from a human strain (G1P[8]) and is administered at 2 and 4 months of age [4–6].

After the World Health Organization (WHO) recommended the inclusion of the RV vaccine in the national immunization programs (NIPs) of all countries in 2009 [7], the worldwide prevalence of RV infection decreased [8, 9]. For example, the hospitalization rate due to RV infection decreased from 11.6 per 10,000 children in 2009 to 1.1 per 10,000 children <5 years of age in 2010 in the United States [8]. The rate of emergency department visits due to RV infection in children <5 years of age also decreased, from 111.1 per 10,000 children in 2009 to 8.3 per 10,000 children in 2010 [8].

In Korea, the Ministry of Food and Drug Safety approved RV5 in June 2007 and RV1 in January 2008, but RV vaccines were not included in the NIP [10, 11]. According to a survey in a secondary hospital in Korea, the proportion of acute gastroenteritis (AGE) due to RV (RV-AGE) during the pre-vaccine period from 2004–2006 was 25.0% in children with AGE, but this decreased to 20.8% in the post-vaccine period between 2008 and 2012 [11]. Although the epidemiologic pattern of RV changed after the implementation of RV vaccination in Korea in 2007, no study has yet analyzed the change in RV infection’s burden or the effectiveness of RV vaccination. Thus, this study aimed to demonstrate the economic burden of RV infection and the change in the prevalence and hospitalization rates for RV infection as a result of RV vaccination after 2007 using National Health Insurance Service (NHIS) claims data in Korea.

## Materials and methods

### National Health Insurance System of Korea and claims data

This study estimated the economic burden of RV infection using a *prevalence-based approach* and estimated the costs due to RV infection from 2009 to 2012 in Korea. The prevalence of RV infection is defined as the number of cases of RV infection in the population (obtained from the NHIS claims data) divided by the population by age (obtained from the Korean Statistical Information Service (KOSIS)) [12–14]. The statistics are reported as rate per 1,000 or 100,000 people [14].

The NHIS was independently established as a single-payer health insurance system in Korea, and the NHIS claims data cover all residents of Korea [15, 16]. We used the International Classification of Disease 10^th^ Revision diagnostic (ICD-10) code A08.0 to identify cases of RV infection used to calculate costs in the NHIS claims database from 2009 to 2012 on the main-disease. Furthermore, to present the sensitivity analysis of prevalence, we estimated RV patients by including the first sub-disease ICD-10 codes and compared this with the main disease in patients.

### Economic burden

The method used to estimate the economic burden of disease was as follows: Economic Burden = Direct costs + Indirect costs (Table 1)

**Table 1:** Classification of economic burden.

Classification	Category	Data Source
***Direct cost***		
Direct medical costs	Outpatient medical cost	NHIS claims data
	Inpatient medical cost	NHIS claims data
	Uninsured medical cost	2012 Survey on the Benefit Coverage Rate of National Health Insurance at NHIS [17].
Direct non-medical costs	Transportation cost	Korea Health Panel Data surveyed at NHIS and KIHASA [18]
	Caregiver cost	Statistics Korea data—2012 Survey Report on Labor Conditions by Employment Type surveyed at Ministry of Employment and Labor [13]
***Indirect cost***		
Productivity loss	Due to morbidity	NHIS claims data
		Statistics Korea data—2012 Survey Report on Labor Conditions by Employment Type surveyed at Ministry of Employment and Labor [13]
	Due to premature death	Statistics Korea Data—Cause of death data in 2012 [13]

NHIS; National Health Insurance Service, KIHASA; Korea Institute for Health and Social Affairs.

#### Direct costs

Direct costs included the direct medical care costs (NHIS-covered and non-NHIS-covered services costs for inpatient, outpatient, and drug costs) and direct non-medical costs (transportation costs). The NHIS data are nationally representative of medical costs in Korea, so we could calculate the number of patients, admission days, and NHIS-covered medical costs using these data [14, 19].

NHIS-covered service costs included the amount paid by the health insurance and the patients’ copayments, while non-NHIS covered services costs (those not covered by the Korean NHIS) were estimated based on the proportion of uninsured service costs using data from the 2012 Survey on the Benefit Coverage Rate of National Health Insurance for medical charges [17]. This purpose of the survey was to investigate the coverage rate of the NHIS, hence, the selected hospitals’ cost data were collected annually.

We estimated transportation costs using the transportation costs from infectious diseases patients from the Korea Health Panel, which provided data on the patterns of health service use and health expenditures [18]. We considered the transportation cost for every outpatient visits and admission cases.

#### Indirect costs

Indirect costs included opportunity costs lost as a result of productivity loss through premature death and morbidity costs. Additionally, caregiver costs were included in the indirect costs. Opportunity costs were estimated via the human capital approach [20]. Productivity loss from premature death due to RV infection was analyzed from the KOSIS raw data for death [13]. We assumed that a person’s economic activity would end at the age of 65 years and estimated the average annual income from the year of death to the year by the age of 64 [15].

Productivity loss for inpatient care was calculated according to the average daily wage by sex and age from KOSIS and the period of inpatient hospital days from the NHIS claim data [12, 13]. Productivity loss for outpatient care was estimated as one-third of the average daily wage [14].

Caregiver costs are the economic losses that result when caregivers stop economic activities to care for patients [15, 21]. Researchers tend to calculate the cost by multiplying the number of visits by the average daily income for a woman aged 50–54 years [15, 21]. However, since about 65% of patients with RV infection were <5 years old, we assumed that mothers primarily took care of the patients [12]. Thus, to determine caregiver costs, we estimated women’s productivity loss based on the averages for women aged 30 to 34 years [13].

For inpatient care, caregiver costs were calculated as the period of inpatient hospital days, and, for outpatient care, the caregiver cost was estimated at about one-third of the daily working hours [14, 19, 22]. Data regarding incomes were obtained from the KOSIS [13]. We conducted sensitivity analysis to assess the robustness of cost estimates. Based on previous studies, we calculated using alternative discount rates (0%, 3%, and 5%) the expected future earning lost due to premature death from the sensitivity analysis [21, 23–25].

All costs were converted into US dollars using the average 2012 exchange rate of the U.S. dollars ($1 = 1126.25 Korean Won) [26]. Statistical analyses were performed using SAS version 9.3 (SAS Institute, Cary, NC, USA).

#### Ethics statement

This study was approved by the Institutional Review Board of Korea University (1040548-KU-IRB-13-164-A-1(E-A-1)(E-A-1)(E-A-1)).

## Results

### Prevalence and days of admission

**Fig 1:**
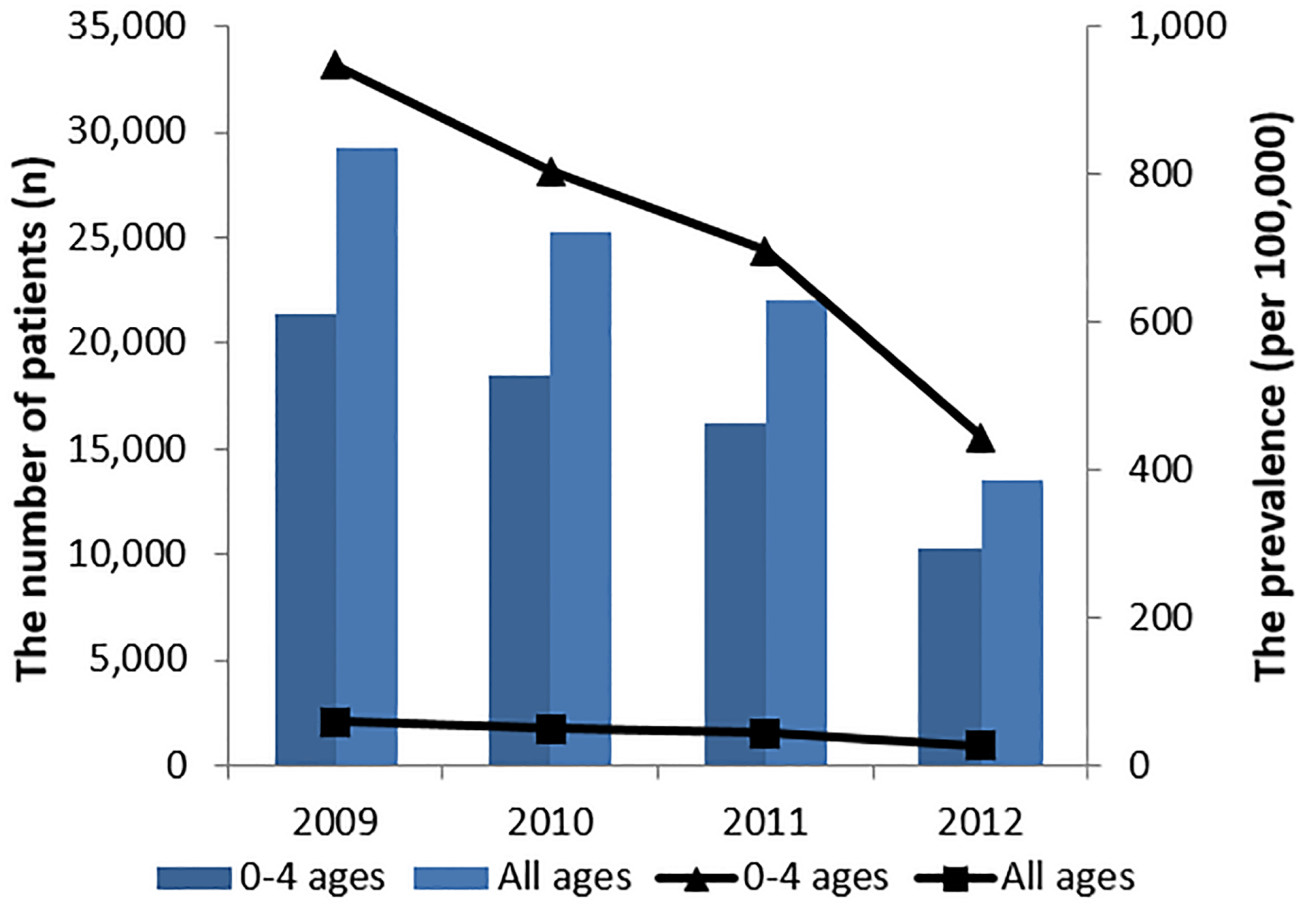
Trend in the prevalence of rotavirus infection over the 4–year period from 2009–2012. The bar graph indicates the change in the number of patients infected with rotavirus from age 0 to 4 years of age and for all age groups. The line graph expresses the change in the prevalence of rotavirus infection in patients aged from 0 to 4 years and for all age groups.

The number of patients <5 years of age with RV infection decreased from 21,437 in 2009 to 18,507 in 2010, 16,215 in 2011, and 10,295 in 2012 (Table 2, Fig 1). The prevalence of RV infection in children <5 years old per 100,000 population also decreased, from 947.10, 804.76, 696.39, and 443.50 in 2009, 2010, 2011, and 2012, respectively (Table 2 Fig 1). The total number and prevalence of RV infections in the overall population in 2012 were less than half of the 2009 prevalence. Since 2009, the overall number of children infected and the prevalence have decreased remarkably (Table 2, Fig 1).

**Table 2:** Prevalence of and mortality rate for rotavirus infection from 2009 to 2012.

Year	Age group (years)	No. of patients	No. of deaths	Prevalence (per 100,000)	Mortality rate (per 100,000)
2009	0–4	21,437	2	947.10	0.09
	5–9	3,670	0	137.99	0.00
	Other	4,157	0	9.27	0.00
	Total	29,264	2	58.79	0.00
2010	0–4	18,507	3	804.76	0.13
	5–9	3,077	0	125.19	0.00
	Other	3,697	0	8.08	0.00
	Total	25,281	3	50.05	0.01
2011	0–4	16,215	1	696.39	0.04
	5–9	2,977	0	126.82	0.00
	Other	2,796	0	6.07	0.00
	Total	21,988	1	43.34	0.00
2012	0–4	10,295	1	443.50	0.04
	5–9	1,932	0	82.30	0.00
	Other	1,312	0	2.83	0.00
	Total	13,539	1	26.57	0.00

The decreasing pattern of RV is also shown when RV cases were included as sub-disease ICD-10 codes (S1 Table).

The overall proportion of RV infections occurring in children aged <4 years old was 73.3% in 2009, 73.2% in 2010, 73.7% in 2011, and 76.0% in 2012; thus, >70% of RV infections occurred in children <5 years old (Table 2). Mortality due to RV infection was relatively small. Only seven children in the overall population died due to RV infection during the study period (Table 2).

Accordingly, the total number of days of admission in children <5 years old decreased, from 76,203 in 2009 to 64,736 in 2010, to 63,536 in 2011, and to 37,707 in 2012. The total number of days of admission for all patients also decreased, from 83,599 in 2009 to 44,024 in 2012 (Table 2). However, there was no remarkable change in the number of days of admission for children aged 5–9 years. In 2010, the number of days of admission increased, from 5,067 in 2010 to 8,310 in 2011, although it decreased to 5,261 in 2012 in this age group (Table 2).

### Economic burden

**Fig 2:**
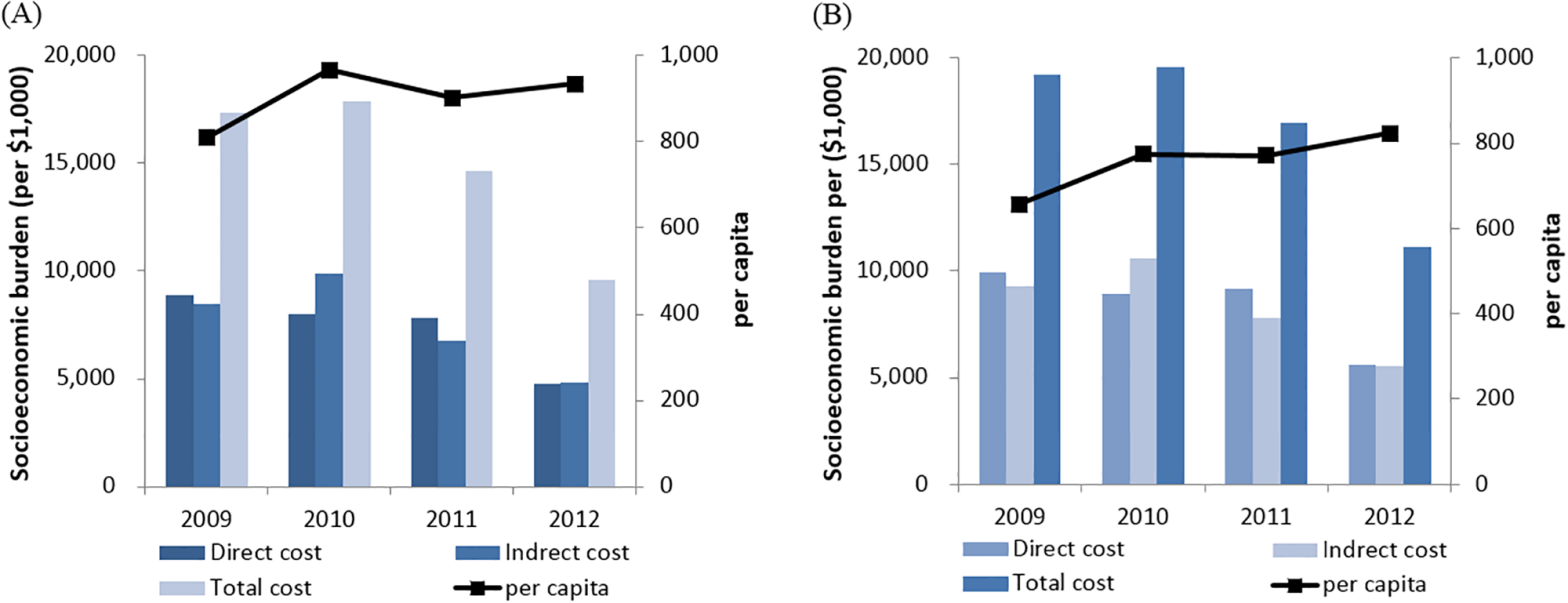
Trend in the socioeconomic burden of rotavirus infection over the 4-year period from 2009–2012. (A) The overall cost and per capita cost for patients under 4 years of age with rotavirus infection. (B) The overall cost and per capita cost for rotavirus infection in patients, in the overall population.

The total costs obtained by summing the direct and indirect costs associated with RV infection in children <5 years old increased from $17.3 million in 2009 to $17.9 million in 2010, to $14.6 million in 2011, and to $9.6 million in 2012 (Table 3, Fig 2A). The total costs in the overall population increased, from $19.2 million in 2009 to $19.6 million in 2010, to $16.9 million in 2011, and to $11.1 million in 2012 (Table 3, Fig 2B). Although the total cost in 2010 was higher compared to that in 2009, the total cost in 2012 had decreased to about half of the cost in 2009.

**Table 3:** Economic burden of rotavirus infection from 2009 to 2012*^†^.

Year	Age group (years)	No. of outpatients	Days of admission	Direct costs	Indirect costs	Total costs	Per capita costs
2009	**0–4**	**13,291**	**76,203**	**8,868,063**	**8,471,304**	**17,339,368**	**808.85**
	5–9	3,472	5,727	776,936	529,096	1,306,032	355.87
	Other	5,038	1,669	310,725	261,161	571,886	137.57
	Total	21,801	83,599	9,955,724	9,261,561	19,217,286	656.69
2010	**0–4**	**12,169**	**64,736**	**7,992,081**	**9,902,621**	**17,894,702**	**966.92**
	5–9	2,848	5,067	695,852	493,780	1,189,632	386.62
	Other	4,577	1,099	252,769	216,870	469,639	127.03
	Total	19,594	70,902	8,940,702	10,613,271	19,553,973	773.47
2011	**0–4**	**8,493**	**63,536**	**7,820,484**	**6,795,758**	**14,616,242**	**901.40**
	5–9	1,864	8,310	1,076,794	784,823	1,861,618	625.33
	Other	3,402	1,341	252,254	216,337	468,591	167.59
	Total	13,759	73,187	9,149,532	7,796,918	16,946,451	770.71
2012	**0–4**	**7,182**	**37,707**	**4,790,216**	**4,823,045**	**9,613,262**	**933.78**
	5–9	1,270	5,261	669,060	545,671	1,214,731	628.74
	Other	1,368	1,056	150,078	156,182	306,260	233.43
	Total	9,820	44,024	5,609,354	5,524,898	11,134,253	822.38

* We estimated women’s productivity loss based on the averages for women aged 30 to 34 years.

^†^ Costs are reported according to the average 2012 exchange rate for the U.S. dollar ($1 = 1125.26 won).

The direct cost for patients <5 years old decreased from $8.9 million in 2009 to $4.7 million in 2012, or by about half (Table 3, Fig 2A). Direct costs in the overall population decreased from $10.0 million in 2009 to $5.6 million in 2012, even though the cost in 2011 ($9.1 million) was higher than the cost in 2010 ($8.9 million) (Table 3, Fig 2B).

Indirect costs for children <5 years old increased from $8.5 million in 2009 to $9.9 million in 2010, but indirect costs in 2011 and 2012 remarkably decreased to less than half of the cost in 2010 ($4.8 million), and the trend related to indirect costs in the overall population was equal to that of children <5 years (Table 3, Fig 2A and 2B).

Changes in per capita costs were also noted to vary with the economic burden of RV infection. Total costs for patients overall decreased from 2009 to 2012, but per capita of patients <5 years old and in the entire population increased from $808.85, $656.99 in 2009; $966.92, $773.47 in 2010, $901.4 and $770.71 in 2011 and to 933.78, 822.38 in 2012, respectively (Table 3, Fig 2A and 2B).

Finally, we conducted the sensitivity analysis using the discount rate of cost. When a 3% discount rate was applied, the total costs were 17.6, 16.6, 16.3, and 10.5 million dollars in 2009, 2010, 2011, and 2012, respectively. When a discount rate of 5% was applied, the total cost decreased to 17.3, 15.9, 16.1, and 10.3 respectively (Table 4).

**Table 4:** The results of sensitivity analysis by discount rate*^†^.

Year	Age group (years)	Total costs		
		Discount 0%	Discount 3%	Discount 5%
2009	**0–4**	**17,339,368**	**15,815,997**	**15,454,085**
	5–9	1,306,032	1,306,032	1,306,032
	Other	571,886	571,886	571,886
	Total	19,217,286	17,693,915	17,332,003
2010	**0–4**	**17,894,702**	**15,003,450**	**14,330,979**
	5–9	1,189,632	1,189,632	1,189,632
	Other	469,639	469,639	469,639
	Total	19,553,973	16,662,721	15,990,250
2011	**0–4**	**14,616,242**	**13,988,287**	**13,832,554**
	5–9	1,861,618	1,861,618	1,861,618
	Other	468,591	468,591	468,591
	Total	16,946,451	16,318,496	16,162,763
2012	**0–4**	**9,613,262**	**8,979,027**	**8,821,737**
	5–9	1,214,731	1,214,731	1,214,731
	Other	306,260	306,260	306,260
	Total	11,134,253	10,500,018	10,342,728

* We estimated women’s productivity loss based on the averages for women aged 30 to 34 years.

^†^ Costs are reported according to the average 2012 exchange rate for the U.S. dollar ($1 = 1125.26 won).

## Discussion

This study estimated the economic burden of RV infection using nationally representative data from the NHIS claims, the Korea Health Panel, and the KOSIS. According to these data, since 2009, the prevalence and admission rates due to RV infection in children have decreased remarkably.

RV infection is primarily spread by children <5 years old [4], and RV vaccines have proven to be highly effective against RV-AGE in children [6]. The efficacy of RV5 against severe G1–G4 RV gastroenteritis was 98.0%, and the efficacy for each serotype was 74.9% (G1), 63.4% (G2), 82.7% (G3), 48.1% (G4), and 65.4% (G9) [5]. Although RV1 is derived from one RV strain (G1P[8]), its efficacy against RV gastroenteritis was 91.8% (G1P[8]), 87.3% (G3P[8], G4P[8], G9P[8]), and 41.0% (G2P[4]) [6]. The RV5 and RV1 vaccines were shown to provide lasting and broadly heterologous protection against RV gastroenteritis in US children between 2012 and 2013 [27]. A German study reported that RV vaccines provided 80% and 68% protection against RV-related hospitalizations and medical treatments among children <30 months old from 2010–2011 [28].

In Korea, RV vaccination also appears to be highly efficacious in protecting against RV-AGE. The Korea Centers for Disease Control and Prevention (KCDC) announced that the group A RV detection rate decreased from 12.3% in 2005 to 5.7% in 2013 for viral AGE cases in Korea [29, 30]. Additionally, the number of infected patients and admission days declined by about half from 2009 to 2012 in children <4 years old, particularly in the current study.

However, the rate of vaccination against RV is still low in Korea. In 2012, the vaccination rate for vaccines included in the NIP, such as BCG and hepatitis B, in 3-year-old children was >90% [31]. At the same time, the rate of first RV vaccination was just 32.3% (second, 31.0%; third, 23.6%–this vaccination rate included RV1 and RV5) per the 2012 Korean National Immunization Survey which is the only source of this immunization rate [31]. These results are likely because the RV vaccination is excluded from the NIP schedule in Korea.

Although the RV vaccination rate remains low, its effectiveness against RV infection is easily observed [11, 32]. Some Korean studies revealed that the incidence of RV-AGE decreased after the introduction of RV vaccine while the RV-AGE infection season was also delayed by about 2–4 months [11, 32]. On the contrary, the rate of norovirus infection in Korean children increased and was the largest cause of viral gastroenteritis in 2010 and 2012 [30]. This result was also observed in the USA, where, after RV vaccines were introduced, norovirus also became the primary cause of medically attended AGE [8]. Thus, the RV vaccine helps to prevent and diminish the severity of RV gastroenteritis, and the economic burden in Korea has decreased due to vaccination.

There were 7 fatal cases in Korean children from 2009 to 2012 in the NHIS claims data. Although fatality cases are rather scarce, the burden of RV is substantial. For example, more than 85% of the total number of days of admission were attributable to young children <5 years old. Thus, the indirect costs related to caregiver costs were a great expense to the Korean society, because many women of working-age needed to take care of their ill children as caregivers [12, 13, 15, 21].

Although the number of outpatients and days of admission decreased, per capita expenditures did not; instead, it showed an increasing pattern from 2009 to 2012, hence, the economic costs of the disease remain a burden for each family and the Korean society.

With respect to government support for childcare, the number of daycare centers in Korea increased about 8-fold since the 1990s (i.e., during the past 20 years) [33]. There were 2,774,066 Korean children <5 years of age in 2013, and the number of children attending daycare centers, including kindergarten, was 2,144,510; thus, the utilization ratio of daycare centers was about 77.3% in children <5 years old [34, 35]. Daycare attendance was a highly significant risk factor for RV infection in children 6–24 months old; thus, RV vaccination is recommended for this age group, particularly for children attending daycare centers [28]. Therefore, increasing the RV vaccination rate is crucial for preventing RV infection and diminishing the economic burden of RV infection in Korea.

The WHO Strategic Advisory Group of Experts (SAGE) on Immunization also recommended adding the RV vaccine to all NIPs, especially in countries in which diarrheal deaths occur in >10% of children <5 years old [36–38].

Since the United Kingdom included the RV vaccine in their national vaccination schedule in July 2013, the rates of attendance and admission for gastroenteritis of any cause reduced by 48% and 53%, respectively in 2014 compared with the mean of cases in the pre-vaccine years 2012 and 2013 [39]. Additionally, the estimated secondary care savings were £7.5 (€10.5) million in the first year after vaccine introduction [39]. A Malaysian study concluded that a universal RV vaccination program could reduce both the disease burden and health inequalities [40]. However, as the vaccination rate of RV is lower than that of vaccinations included in the NIP in Korea, the RV vaccination should be added into the NIP for Korean children.

This study has some limitations. First, a diagnosis code was used to calculate the number of cases in this study, but RV tests were not performed for all gastroenteritis patients. Thus, there is a difference between the number of patients in our study and the overall number of patients in Korea. Additionally, as gastroenteritis can sometimes be caused by a mixed pathogen [41], estimation of the sole economic burden of RV infection is limited. Also, patients with mild RV gastroenteritis could be nursed by their caregivers at home without visiting the clinic. The claims data did not capture such types of RV, and this could also result in an underestimation of the RV burden. Similarly, because the claims data are originally intended for insurance claims, the accuracy of ICD-10 codes could be biased compared to medical records [14]. Finally, though a cost-effectiveness analysis could analyze the necessity of RV vaccination precisely, we did not perform cost-effectiveness analysis due to limited data availability. This kind of economic analysis needs to be conducted in the future.

In conclusion, after RV vaccines were introduced in Korea, the number of patients and the economic effects of RV infection decreased. However, indirect costs with respect to the loss of caregiver’s productivity increased because the infection was mainly found in children aged <5 years and per capita expenses showed an increasing pattern. Therefore, including the RV vaccination in the NIP would contribute to reducing the prevalence of RV infection, economic loss from RV infection, and improving the quality of life in Korean children in the long-term.

## Supporting information

S1 Table Prevalence for rotavirus infection including first sub-disease ICD-10 codes from 2009 to 2012. (DOCX)
