# Supplementary material for: The economic burden of rotavirus infection in South Korea from 2009 to 2012
Source: PLoS One. 2018 Mar 19;13(3):e0194120. doi: 10.1371/journal.pone.0194120 (PMC5858784; doi:10.1371/journal.pone.0194120)
Supplement: S1 Table — (DOCX) [file pone.0194120.s001.docx]

**Supporting information caption**

**S1 Table. Prevalence for rotavirus infection including first sub-disease ICD-10 codes from 2009 to 2012**

| Year | Age group (years) | No. of patients | Prevalence (per 100,000) |
| --- | --- | --- | --- |
| 2009 | 0–4 | 28,713 | 1268.6 |
|  | 5–9 | 5,122 | 192.6 |
|  | Other | 6,016 | 13.4 |
|  | Total | 39,851 | 80.1 |
| 2010 | 0–4 | 25,044 | 1089.0 |
|  | 5–9 | 4,133 | 168.2 |
|  | Other | 5,302 | 11.6 |
|  | Total | 34,479 | 68.3 |
| 2011 | 0–4 | 21,748 | 934.0 |
|  | 5–9 | 3,810 | 162.3 |
|  | Other | 4,415 | 9.6 |
|  | Total | 29,973 | 59.1 |
| 2012 | 0–4 | 14,132 | 608.8 |
|  | 5–9 | 2,493 | 106.2 |
|  | Other | 1,863 | 4.0 |
|  | Total | 18,488 | 36.3 |
